# Nutritional Factors Related to Canine Gallbladder Diseases—A Scoping Review

**DOI:** 10.3390/vetsci12010005

**Published:** 2024-12-28

**Authors:** Fabio Alves Teixeira, Kathleen Moira Aicher, Ricardo Duarte

**Affiliations:** 1 School of Veterinary Medicine and Animal Science, University of São Paulo-Brazil, São Paulo 05508270, Brazil; 2 Gastrointestinal Laboratory, Department of Small Animal Clinical Sciences, Texas A&M University, 4474 TAMU, College Station, TX 77843-4474, USA; 3 Gastrovet, São Paulo 04077003, Brazil

**Keywords:** canine, biliary sludge, mucocele, cholelithiasis, biliary obstruction, hyperlipidemia, endocrinopathies, cholesterol, fat, hepatic disease

## Abstract

Gallbladder mucocele, cholelithiasis, choledocholithiasis, and cholecystitis are significant contributors to morbidity and mortality in dogs. The exact etiology of these conditions remains poorly understood, though various factors, such as endocrinopathies, dyslipidemia, and impaired gallbladder motility, have been suggested as potential contributors. Surgical intervention has been described as the first choice of treatment when biliary rupture or obstruction is suspected; however, medical management may be an important part of therapeutic or preventative strategy. Reports of medical management typically involve the use of a choleretic used to stimulate the flow of bile into the duodenum or substances that act as a “hepatoprotective” agent such as S-adenosylmethionine. In people, some nutrients appear to modify bile flow and are used as agents in the prevention and treatment of these conditions in the gallbladder. This paper provides a review of the literature about possible nutritional factors involved in the pathogenesis and treatment of canine gallbladder mucocele and cholelithiasis. Opportunities for the prevention and treatment of common biliary diseases in dogs may include the reduction of dietary fat, control of hyperlipidemia with omega-3 and fiber supplementation, ensuring an adequate supply of amino acids such as methionine and tryptophan, and the evaluation of vitamins such as vitamin D.

## Simple Summary

Gallbladder diseases in dogs are becoming increasingly common and can lead to extrahepatic bile duct obstruction or biliary rupture, resulting in jaundice, vomiting, abdominal pain, peritonitis, and even death. Issues related to treatment and prevention need to be discussed. Decades ago, dogs were used as a model to study the formation of cholelithiasis in humans, with a focus on nutritional aspects. After a period of limited research, this topic has gained attention again in recent years, particularly in veterinary medicine literature. Nutritional factors are once again being explored. However, no paper comprehensively summarizes the existing literature in this field. Understanding the role of nutrition may provide valuable insights for the clinical management of dogs with gallbladder disease and guide future research. This article reviews the role of nutrition in gallbladder physiology, with an emphasis on preventive and therapeutic approaches for modifying bile fluidity (e.g., mucocele) and preventing gallstone formation. Key nutrients involved in these processes include fats, possibly omega-3 fatty acids with a nutraceutical effect, and essential amino acids such as methionine and tryptophan, which play a role in basic physiological functions.

## 1. Introduction

Bile contains water, electrolytes, cholesterol, phospholipids, hormones, protein, bilirubin, and other substances that are excreted into the feces. Bile acids are the major constituents of bile, accounting for approximately one-half to two-thirds of the total solutes. Under normal conditions, the mucosa of the gallbladder can reabsorb compounds such as water and electrolytes in order to concentrate the bile [1].

Studies have shown that the incidence of canine gallbladder diseases, including biliary sludge, gallbladder mucoceles, and gallstones, have increased, frequently presenting as a life-threatening condition [2]. While biliary sludge is not considered a disease in itself [3] and cholelithiasis may sometimes represent ultrasound findings without apparent clinical relevance [4], gallbladder mucoceles and cholelithiasis are considered the most common and important extrahepatic biliary condition in dogs, and may lead to gallbladder obstruction or rupture, and consequently bile peritonitis [5]. Gallbladder mucocele formation in dogs is a condition characterized by the excessive secretion of thick mucus by the gallbladder epithelium, leading to the potential obstruction or rupture of the gallbladder. According to the literature, the first description of a canine gallbladder mucocele-like case was published in 1965 [6], followed by only a few isolated reports [7,8]. In 1995, a publication [9] formally diagnosed their case as a gallbladder mucocele.

Dogs with biliary obstruction may have emesis, anorexia, abdominal pain, jaundice, leukocytosis, fever, elevated serum liver enzyme activity, and hyperbilirubinemia. The condition is considered serious due to the high associated mortality rates [10,11,12,13,14]. Recently, a study showed that poor appetite may be a sign that can help to identify dogs with early stages of gallbladder disease [15,16].

The pathophysiology of gallbladder mucocele and cholelithiasis remains poorly understood [17]. In one study, the authors state that “the underlying cause of gallbladder mucocele formation in the dog is currently somewhat of a mystery” [18]. Studies in other species [19] and clinical evaluations of sick dogs [20] suggest that alterations in the composition and motility of bile and mucoid content might be considered risk factors for calculogenesis and may contribute to the occurrence of biliary sludge and gallbladder mucocele [18,21,22].

**Figure 1:**
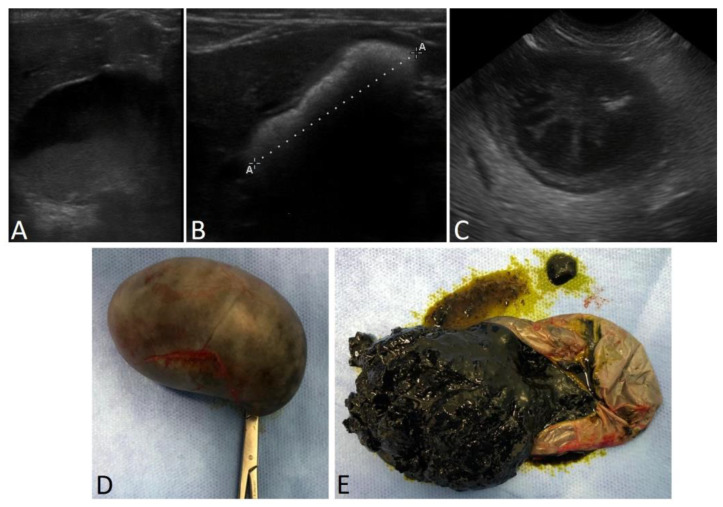
Canine gallbladder conditions. (**A**) Ultrasound image of biliary sludge. (**B**) Ultrasound image of a gallbladder almost entirely filled by cholelithiasis. (**C**) Ultrasound image of gallbladder mucocele. (**D**) Photograph of a gallbladder after cholecystectomy indicated due to the risk of gallbladder rupture (**E**) and the same gallbladder after wall incision.

In humans, it has been proposed that altered gallbladder motor activity precedes the formation of gallstones and gallbladder mucocele [23]. Alterations in biliary motility and fluidity are increasingly recognized in dogs [14,17,24]. These alterations have been proposed to be associated with the formation of biliary sludge (higher bile viscosity, resulting from the slow settling of particles dispersed; Figure 1A), gallbladder mucocele (progressive accumulation of mucin-laden bile and hyperplasic biliary mucosa; Figure 1C–E) and cholelithiasis (Figure 1B) by increasing the chances of developing cholesterol, calcium, and bilirubin precipitates into crystals and lithiasis [17,21,25].

Although cholelithiasis, biliary sludge, and gallbladder mucocele may share common events in their formation, each one of those conditions may have a unique, and perhaps multifactorial etiology. In one study, dogs with biliary sludge were followed by ultrasound for a period of one year and none of them developed gallbladder mucocele or cholelithiasis [26]. However, in more recent investigations, the authors suggest that biliary sludge is not a benign condition, as the odds of developing gallbladder mucocele were higher in animals with non-gravity-dependent biliary sludge [27]. Mucins are the major components in biliary sludge and gallbladder mucoceles, suggesting a similar pathogenesis, however they differ in cholesterol content [17,28].

From a therapeutic standpoint, especially for gallbladder mucoceles, while medical management is associated with shorter survival compared to surgical treatment, it may be a reasonable alternative when surgery cannot be pursued [29]. Drugs that reduce hyperlipidemia, stimulate the flow of bile, or act as liver antioxidants can be employed; however, the treatment of choice is cholecystectomy [2,29,30]. Studies to prospectively investigate medical management of biliary disease in dogs are lacking, and are largely guided by patient and client factors, as well as experiences of the attending a veterinarian. Financial limitations of the client, as well as patient risk factors such as comorbidities or anesthetic risk may preclude surgery in many cases. Therefore, treatment and prevention alternatives for canine biliary disease are essential.

Researchers have proposed that diet may play a role in the pathogenesis and treatment of gallbladder mucoceles and cholelithiasis in human biliary disease [31,32,33,34]. Similarly, reports in veterinary studies document the reversal of these conditions in dogs following medical management that includes targeted nutritional interventions [11,30].

The objective of this article is to provide a narrative review focusing on the relationship between nutritional factors and canine gallbladder diseases, such as gallbladder mucocele and cholelithiasis.

## 2. Dietary Fat Content and Hyperlipidemia

Only one study was found that compares two diets in the same experimental design with the objective of evaluating gallbladder emptying time [35]. The authors conducted a crossover study, with seven healthy dogs fed a high-fat-high-cholesterol diet and then a commercial low-fat therapeutic diet (8.5% fat, in dry matter basis—data obtained on company website information visited on 9 March 2019) for 2 weeks per each diet, with a 4-month washout period in between diets. The high-fat-high-cholesterol diet was prepared by adding 10% (wt/wt) beef tallow and 10% (wt/wt) cholesterol to a commercial maintenance diet, resulting in 20% protein, 20% fat, 8% cholesterol, 29% starch, and 6% dietary fiber. In this study, the concentration of total cholesterol in plasma and gallbladder bile increased after high-fat-high-cholesterol diet feeding. Also, when compared with feeding of the basal diet, the high-fat-high-cholesterol diet caused the gallbladder bile acid composition to be more cytotoxic with resultant gallbladder hypomotility, as observed by decreased gallbladder emptying after induction of its gallbladder motility by cholecystokinin infusion. The authors attributed the decrease in gallbladder cholecystokinin sensitivity and modifications in the gallbladder bile acid composition to the higher cholesterol concentration in the bile and plasma.

Another research group induced gallstone formation in healthy dogs using a specific diet with a cholesterol inclusion (1%) [36]. The conversion of cholesterol to bile acids represents an important route by which cholesterol is excreted [37,38]. Thus, high cholesterol consumption could result in higher excretion of biliary cholesterol, increasing the risk of cholesterol crystal aggregation and precipitation.

Using dogs as experimental models, earlier studies evaluated the impact of normal diets fed to healthy dogs plus cholesterol and observed increased concentrations of plasma and bile cholesterol and a modified bile acids profile [38,39]. In addition, high biliary cholesterol and changes in the bile acid profile could be linked to changes in cholecystokinin receptors, which may in turn impact the motility of gallbladder [40,41]. As for other mammals, there is a speculation that high bile cholesterol concentrations cause gallbladder hypomotility and create a permissive environment allowing normal concentrations of hydrophobic bile salts to inflame the gallbladder mucosa and impair muscle function, inhibiting gallbladder emptying [40].

Although the studies of diets supplemented with cholesterol in dogs were important steps to understand the relationship between diet and the prevention or treatment of gallbladder disease, the authors did not supply detailed dietary information, such as nutritional composition and ingredients [35,36,38,39,41,42,43,44,45]. In addition, dogs in different treatment groups within the same study did not consume the same amount of calories during each experimental diet period. In studies where the authors used a lithogenic diet (LD) to induce gallstone formation [36,41,43,45,46], there were more diet modifications than cholesterol inclusion, which could explain the biliary alterations. Therefore, previous data suggested that healthy dogs have compensatory mechanisms to maintain normocholesterolemic in the face of a high cholesterol intake [47,48], with one recent study reporting no difference over time in plasma and bile cholesterol concentration after discontinuation of a cholesterol-rich diet in dogs [49]. Thus, in an attempt to avoid gallbladder disease, we cannot conclude that fat should be restricted in healthy dogs, since ingestion of fatty acids appears to be important in the stimulation of cholecystokinin secretion [50], neither can it be affirmed that cholesterol should be restricted in dog diets. Nonetheless, these restrictions might be important to animals with a predisposition to high blood cholesterol concentration, as well as animals with endocrinopathies. It has been shown previously that hormones can modify gallbladder metabolism [51]. Previous studies demonstrated that glucocorticoids down-regulate cholecystokinin gene expression in the rat intestinal mucosa and in two cholecystokinin-producing cell lines [52]. Endocrinopathies are considered as an important predisposing factor [53,54], with the odds of mucocele in dogs with hyperadrenocorticism being 29 times that of dogs without it [55]. However, high doses of corticosteroids provided to dogs in an experimental study failed to induce any changes consistent with gallbladder mucocele [56].

Studies have revealed associations between hyperlipidemia and certain endocrinopathies associated with a higher incidence of gallbladder mucocele [5,11,12,55,57,58,59,60] and cholelithiasis in dogs [61]. Dogs with hypercholesterolemia had a high odds ratio (OR) to present with gallbladder mucocele (OR = 2.92) and cholelithiasis (OR = 9.72) [58,61]. Also, hypertriglyceridemia was shown to be a risk factor for cholelithiasis (OR = 12.91) and for gallbladder mucocele (OR = 3.55) [58,61] in dogs. Frequently, hyperlipidemia in dogs is secondary to endocrinopathies [62], which in themselves alter the viscosity of bile [25] and metabolism of bile acids [63,64] and may predispose to gallbladder mucocele formation [54,55,61]. A recent study showed that hyperlipidemic dogs have a significantly greater fasting and postprandial gallbladder volume compared to control dogs [65]. The authors suggested that gallbladder distention could contribute to the retention of bile and potentially the development of gallbladder disease.

Some researchers have shown a reduction in blood lipid metabolites after increased fiber intake in dogs with endocrinopathies [66,67]. This effect can be explained by increased fecal cholesterol and bile acids excretion associated with stimulating synthesis of new bile acids and cholesterol catabolism [68]. The impact of fiber in blood triglycerides concentration could be related to the reduction in fat digestibility, which could result in lower triglyceride absorption and postprandial hypertriglyceridemia. However, other authors have observed that increasing fiber in canine diets resulted in little or no effect on fat digestibility [69,70,71,72,73].

Regarding dietary fat content, one study evaluated hyperlipidemic diabetic dogs and found that diets with lower levels of ether extract resulted in lower plasma concentrations of cholesterol, free fatty acids, and glycerol [74].

However, low-fat diets may not be suitable for all patients, as some animals on high-fiber, low-fat regimens may experience a reduced appetite, resulting in weight loss or inadequate weight gain. Additionally, these diets can lead to an increased volume of softened feces, excessive flatulence, constipation, vomiting, and dull or opaque hair [66,67,74].

Changes in starch sources can be a tool to aid with control of hyperlipidemia. Recent research found that peas and barley as exclusive starch sources could result in lower plasma triglycerides and cholesterol concentrations than corn-based diets when fed to hyperlipidemic and diabetic dogs [75]. Studies with humans found similar results and attributed lower blood cholesterol concentrations to the indirect effects of nutrition on gallbladder physiology. Higher consumption of pea fiber could promote higher excretion of bile acids due to a direct effect on intestinal transit time and an increase in bile acids in the feces [76].

β-glucan from barley is also associated with reduced gastric emptying, digestion, and absorption of cholesterol and fat, and increased excretion of bile acids, neutral sterols, and catabolism of cholesterol [77,78]. Some researchers correlated the consumption of pea protein with up-regulation of the enzymes of the bile acid synthesis pathway and an increase in the hepatic LDL-receptor mRNA concentration, accelerating clearance of LDL-cholesterol particles and increasing excretion of bile acids via feces [79,80,81].

In the literature, there is a recommendation to supplement hyperlipidemic dogs with fish oil to increase intake of omega-3 polyunsaturated fatty acid, mainly eicosapentaenoic and docosahexaenoic acids [62,82]. The lipid-lowering effect of omega-3 fatty acids was observed in healthy adult [83,84,85,86,87], mature [88,89], and hyperlipidemic dogs [90]. However, supplementation with fish oil did not have a significant effect on concentrations of cholesterol and triglyceride in other studies [91,92,93,94,95], probably due to the relatively low-dose, short period of supplementation, or high omega-6:omega-3 ratio used in these studies. Bauer [82] recommended that approximately 120 mg of EPA and DHA/body weight (kg)^0.75^ on a metabolic body weight basis be administered in combination with a low-fat diet and that a subsequent reevaluation take place after 6 to 8 weeks of therapy.

Thus, the dietary omega-3 fatty acid-induced decrease in serum triglycerides and cholesterol may prove beneficial in dogs with hyperlipidemia. Some mechanisms have been proposed to explain this phenomenon, such as modifications of intestinal gene expression, agonist action, on nuclear transcription factors, like PPAR-α e PPAR-γ, and lower production of inflammatory markers and of specific G protein ligands that alter certain metabolic cascades, including lipoprotein metabolism [96,97,98]. Omega-3 fatty acids seem to act directly on gallbladder emptying time in humans [99,100,101] and rodents [102,103], but this has not been tested in dogs. In the rodent study, it was observed that dietary supplementation with omega-3 fatty acids can decrease the arachidonic acid (omega-6) content of phospholipids in gallbladder mucosa and bile, resulting in decreased prostaglandin synthesis [102], and prostaglandins can stimulate mucin secretion in canine gallbladder epithelial cultured cells [104,105]. In addition to the effect on hyperlipidemia, a recent study observed that dogs with mucocele, compared to age- and breed-matched healthy dogs, had a lower abundance of EPA and DHA [106]. This could suggest the dietary increase of omega-3 in dogs’ diets as a preventive or therapeutic approach for mucocele, although this still requires further investigation. In this study, plasma lipid abnormalities and histochemical and ultrastructural examinations of the gallbladder mucosa from dogs with gallbladder mucocele formation and control dogs were investigated, reinforcing the hypothesis of lipid metabolism alterations being involved in the disease pathogenesis [106].

Obesity could also be associated with a higher occurrence of gallbladder disease, as obese dogs have higher serum leptin concentrations [107,108,109,110,111,112]. Elevated blood leptin concentration is associated with hyperlipidemia and cholelithiasis, and the gallbladder of dogs with mucocele has more receptors for this adipokine [61,113,114]. Moreover, there is evidence in humans that metabolic changes resulting from weight loss impact the risk of biliary sludge formation and cholelithiasis [115], an aspect not yet studied in obese dogs.

## 3. Protein and Amino Acids

Protein and amino acid ingestion is known to alter gallbladder metabolism. The secretion of hormones related to gastrointestinal tract motility and contraction of its smooth muscle is altered by dietary content of protein and amino acids [50,116]. It is suspected that the protein source can increase biliary output after ingestion of meat products by dogs [117], and the amino acid tryptophan apparently stimulates bile flow. Indeed, supplementation of a gelatin-based diet with tryptophan increased the bile salt secretion in dogs to levels close to that observed with a meat products-based diet, while dietary tryptophan deficiency resulted in lower bile flow [118].

In a series of studies attempting to induce gallstone formation by feeding dogs with an LD, it was evident that methionine influences gallbladder metabolism to the point that a deficiency of methionine modifies bile acid content and stimulates gallstone formation [36,41,43,45,46]. The LD was composed of purified casein (10%), sucrose (50%), corn starch (26%), animal lard (5%), cholesterol (1%), salt mixture (5%), minerals (2%), and cellulose (1%) [36]. Based on current adult canine nutritional recommendation from the National Research Council (NRC) [119], LD exceeded all nutritional requirements, except for methionine, and was at the limit for protein for protein. The dietary profile of those studies, on a caloric basis (/100 kcal), compared to NRC [119], was: 2.5 g of protein (equal to the recommended allowance); 1.3 g of fat (below recommended allowance: 1.38); 18.7 g of carbohydrate (no canine requirements); and 56 mg of methionine (below the minimum requirements: 65 mg).

The LD diet was used based on the following premises: (i) The LD diet alters biliary content (phospholipids, cholesterol, bile pigments, and bile acids), resulting in lower bile secretion and more bile stasis. According to previous canine research, there is a decrease in the production of endogenous bile acids due to protein catabolism caused by a protein deficient diet, aggravated by high consumption of simple sugars, with canine bile flow lower than fasting moments [117,120,121]; (ii) a reduced amount of dietary unsaturated fat, mainly linoleic and arachidonic fatty acids, alters cholesterol-binding capacity in the human gallbladder, resulting in high free cholesterol that can precipitate and aggregate [99,122]; and (iii) high cholesterol intake can exceed the binding capacity of cholesterol in the gallbladder and increase its availability for gallstone formation [36].

In the studies that used the LD diet, dogs developed concretions that adhered to the gallbladder mucosa measuring between 5 to 6 mm, progressing in size, approximately one week after receiving the diet [36,41]. In addition, after administering the LD diet, the dogs showed changes in the bile acid profile and increases in bile mucin concentration [44], akin to those observed in gallbladder mucocele pathogenesis [17,21].

In another study that applied LD [41], six dogs received the diet for twelve months, while another six dogs were fed with a normal diet indicated for adult healthy dogs for the same period. Dogs who received LD had higher measurements of blood cholesterol and lower serum taurine concentrations. Regarding the bile profile, they had lower amounts of bile acids and taurine, and higher amounts of phospholipids, free cholesterol, and free bile acids. Macroscopy and histology of the gallbladder showed signs of greater secretory activity in animals under LD. The authors associated the taurine deficiency in dogs with decreased sulfur amino acid intake due to the lower amount of methionine in LD. Since taurine acts in the conjugation of bile acids, its biological reduction may have contributed to the lower solubilization of compounds in bile, such as cholesterol.

Therefore, it would be reasonable to assume that taurine supplementation could be a candidate as a strategy to prevent gallbladder diseases. However, Christian et al. [45] compared the effects of four diets: control; LD; LD plus taurine (45–55 mg/kg body weight/day); and LD plus methionine (45–55 mg/kg/day). The control diet did not cause changes in the gallbladder. However, LD generated biliary sludge, changes in the bile acid profile, increased bile concentration of mucin and calcium, along with cholelithiasis in all dogs and lower bile and hepatic concentrations of taurine. Dogs supplemented with taurine maintained adequate hepatic taurine concentrations, but demonstrated similar changes as dogs with LD without taurine supplementation including reduced biliary taurine and hepatic methionine levels and the presence of lithiasis.. Among the dogs supplemented with methionine, there were minor changes in the gallbladder, with only one dog having small microliths, and maintenance of the hepatic concentrations of taurine and methionine compared to controls. The “gallbladder protective” effect of methionine ingestion was attributed to its participation in free radical neutralization reactions, modulation of neural and muscular excitability and hormone release, control of calcium entry into cell membranes, and its function as a precursor for the synthesis of homocysteine, glutathione, and S-adenosylmethionine (SAMe). The latter compounds act as liver antioxidants, in methylation and transulfuration reactions, which explains the indication of SAMe in the treatment of liver diseases, including gallbladder mucocele [21,29,30,123,124].

Thus, methionine may inhibit changes in the gallbladder, such as thickening and biliary stasis secondary to toxicity, and maintain resorption and secretion of compounds in the gallbladder. Despite the low chance of methionine deficiency in commercial foods, it is considered a limiting amino acid in the canine diet formulation process, with a possible need for the inclusion of purified DL-methionine [119]. Moreover, earlier studies reported the occurrence of methionine and tryptophan deficiency in homemade diets in 10.1 to 71.8% and in 6.0 to 51.3% [125,126,127,128] and possibly also in vegetarian and vegan canine diets [129,130]. Of important note, there is evidence of possible methionine intoxication in dogs [131], suggesting that guaranteeing the recommended amount is imperative to avoid either deficiency or intoxication [119].

Although low protein and amino acid intake are related to changes in the gallbladder, there are no clear recommendations for maximum protein intake. Recently, a study showed an association between gallbladder mucocele formation and proteinuria in dogs [132]. That study was not designed to comprehensively investigate underlying causes of proteinuria in dogs with gallbladder mucocele; however, if the proteinuria in these dogs is of a renal cause, the recommendation would be to strategically limit protein intake rather than increase it [133].

Regarding protein, a recent study showed the possibility of a relationship between gluten sensitivity and the occurrence of gallbladder mucocele, characterized by reduced serum cholecystokinin and increased transglutaminase-2-IgA autoantibodies in Border Terriers with gallbladder mucoceles [134], which is in part homologous to gallbladder disease identified in human coeliac disease [135]. However, it was specific to Border Terriers, a breed with known gluten sensitivity as the cause for paroxysmal gluten-sensitive dyskinesia [136]. Only one study could be found that described providing a hypoallergenic diet for dogs with biliary sludge. However, numerical measurement of the volume and ejection fraction only took place after the dietary test period, making it impossible to assess the effect of this diet on gallbladder parameters [16].

## 4. Micronutrients

Although methionine supplementation inhibited the development of lithiasis, this amino acid has not been able to prevent the increase in bile calcium concentration [45], which has been observed in other studies [43,46]. This reinforces the hypothesis that calcium acts in the pathogenesis of cholelithiasis [42,137]. While in humans there is evidence that calcium deficiency is related to cholelithiasis, there is concern that short-term calcium supplementation may be deleterious in this context. Calcium has a role in the chelation of cholesterol and bile acids in the gut and in acting in the reduction of bile cholesterol concentration [33], but studies in other species revealed that short-term calcium supplementation influenced bile composition and is related to gallstone formation [138,139].

A previous study with dogs reported a correlation between high blood calcium and high bile calcium [137]. Although those studies with dogs shown increased concentrations of bile calcium [42,43,46,137], they provided little information on mineral content in the test and control diets, which precludes further discussion of the possible relationship between dietary calcium and pathogenesis of cholelithiasis and gallbladder mucocele.

The most recent study found in the literature with information on nutrients and gallbladder disease showed a possible correlation between gallbladder mucocele and vitamins. The study compared healthy dogs and dogs with gallbladder mucocele through metabolomic analysis and found three to ten times lower concentrations of pantothenate, riboflavin, and niacin in the bile of diseased dogs [140]. Additionally, a study in humans highlighted the importance of pyridoxine in gallbladder diseases, as this vitamin participates in the conversion of linoleic acid to arachidonic acid, hence in the increase of the binding capacity of biliary cholesterol [99]. In addition to the possible hypolipidemic effect of niacin [62,141], no other clear description of the relationship between these water-soluble vitamins and the canine gallbladder metabolism has been found in the literature.

Also, some authors in veterinary medicine recommend the supplementation of fat-soluble vitamins in cases of gallbladder mucocele and cholelithiasis, due to the chronic cholestasis that may occur in these cases, which could result in poor assimilation of dietary fat [142]. A previous study showed that the absence of normal bile flow into the intestinal lumen led to a progressive depletion of vitamin D pools. Those authors suggested that vitamin D depletion occurred by factors such as secondary malabsorption of the vitamin due to the absence of adequate amounts of bile salts in the intestinal lumen [143]. A recent study showed that dogs with gallbladder mucocele have lower serum calcidiol concentrations than healthy dogs and that decreasing serum vitamin D concentrations was associated with a more advanced developmental stage of gallbladder mucocele [144]. In that study, the authors did not assess if reduced vitamin D is a cause or effect of biliary disease and did not investigate if vitamin D supplementation could be beneficial for dogs with gallbladder mucocele. Importantly, excessive vitamin D supplementation should be avoided as it was observed to be linked to high bile calcium concentration after dogs received ergosterol supplementation [137].

Finally, the relationship between the dietary profile of dogs and the changes in gallbladder content was found in one study [59], in which researchers evaluated ultrasound images of 1021 dogs and found a higher frequency of biliary alterations among animals older than 10 years, as observed in other studies [3,25]. They reported a higher odds ratio of biliary sludge in dogs exposed to treats [145] like rawhide chews and biscuits, with no correlation found with frequency of feeding or with intake of human food and of homemade food, commercial feed, or a combination of both [59]. In the study, the exact nutritional profile received by the animals was not evaluated, which made it impossible to further analyze the relationship between nutrients and the occurrence of biliary alterations in the clinical patients.

## 5. Feeding Plan and Gallbladder Emptying

First, it is important to understand the effect of feeding on gallbladder physiology. In the absence of stimuli, as in fasting, the gallbladder remains relaxed, and its distal sphincter contracted. The bile content is stored and, due to the continuous reabsorption of water and electrolytes through the mucosa, it becomes thicker. In the postprandial phase, neural (acetylcholine) and hormonal (cholecystokinin) mechanisms stimulate gallbladder contraction, sphincter relaxation, and consequently gallbladder emptying [22]. Thus, in fasting periods, bile tends to be less fluid. In humans, studies have shown increased risk for gallbladder disease when patients undergo prolonged fasting, including during sleep time [32,146,147,148,149]. A study with humans demonstrated the importance of how meal nutrients act with hormone stimulants to affect gallbladder motility. Effects on cholecystokinin secretion were compared between subjects fed liquid diets (1.5 kcal/mL, consisting of 40% fat, 20% protein, and 40% carbohydrate), with subjects fed a control diet (400 mL of either water, 0.9% sodium chloride or 2% sodium chloride). Cholecystokinin secretion stimulation was observed only after feeding with the diet containing macronutrients [50]. This study showed the relation between cholecystokinin and decrease of gallbladder volume. A recent study [150] showed that seeing or smelling food did not increase serum cholecystokinin or serum bile acids concentrations in healthy dogs, suggesting that these factors are unlikely to play a role in gallbladder emptying. 

In some studies, the pattern of canine gallbladder motility in response to feeding or fasting was detailed, finding similarities to that observed in humans [151], showing that keeping dogs fasted decreased the flow of bile [120,121], and a meal resulted in the release of 5% to 65% of gallbladder bile [152]. Normal post-prandial gallbladder ejection fraction in dogs has been described to range from 25% to 60% [16,151,153,154,155]. However, there is no evidence of an association between fasting and the development of gallbladder diseases in dogs.

In an attempt to stimulate gallbladder emptying, Ramstedt et al. [154] compared the effects of diets with and without erythromycin (motility stimulant in the gastrointestinal tract) in healthy dogs, using two doses (1.0 and 2.5 mg of erythromycin/kg of body weight). The authors found no differences between the three treatments, which allows us to infer that the diet used [7.4 g protein and 6.1 g fat (/100 kcal)] had the same stimulating effect in gallbladder emptying as erythromycin.

There are a few studies describing diet features and the release of gallbladder bile. Jonderko et al. [153] obtained a mean of 39.1% of gallbladder emptying at a timepoint of 30 min after feeding and 77.6% at a timepoint of 120 min after feeding dogs with a moist diet consisting of 35.5% protein (dry matter basis), 20.7% fat, 31.8% of carbohydrates, and 12.0% of mineral matter. In another study, Solórzano [156] provided healthy dogs with a moist diet with 44.3% protein, 30.2% fat, 17.0% non-nitrogenous extractives, and 0.4% fiber, and obtained 47.8% emptying at two hours postprandial. Comparing the dietary profile between these studies is difficult due to variations in the methodologies used and a lack of details about the diets used. Nevertheless, the food with the highest fat content resulted in a lower percentage of gallbladder emptying after a two-hour period.

## 6. Clinical Summary

There is substantial evidence suggesting that nutritional interventions can modulate gallbladder function and influence the course of gallbladder diseases in dogs. The studies consistently reported that high-fat diets are associated with increased risk of gallbladder disease, while diets low in fat and supplemented with omega-3 fatty acids and fiber may reduce this risk.

Despite the prevalence of studies conducted on healthy dogs and the lack of detailed diet information in most publications, some nutritional factors appear to be related to the pathogenesis, prevention, and even treatment of gallbladder mucocele and cholelithiasis. The main evidence was the reversal of biliary alterations in all animals and dissolution of the gallstone in 50% of dogs under the LD diet, six weeks after switching from a complete and balanced diet indicated for healthy adult dogs [36,41,43,44,45,46].

In addition, there are reports of success in the clinical treatment of gallbladder mucocele, with dietary switching to low-fat diets associated with SAMe (related to methionine metabolism) and choleretic drugs such as ursodeoxycholic acid [11,29,30]. Thus, the diets prescribed as adjuvant therapy in cases of gallbladder mucocele and cholelithiasis, or predisposition to these conditions, should be complete [119], with adequate vitamins and protein content, especially methionine and tryptophan, moderate amounts of fat with special attention to dietary cholesterol, high dietary fiber content, without excesses in calcium, and with appropriate supplementation of omega-3 fatty acids in order to prevent or control hyperlipidemia.

Regarding nutritional management, control of caloric intake is fundamental to avoid excessive weight gain and comorbidities known to be associated with obesity. It should also be recommended that dogs predisposed to gallbladder mucocele or cholelithiasis receive more frequent feeding, without periods of prolonged fasting. Moreover, further studies regarding cholesterol ingestion in dog nutrition are needed to better understand the role of dietary cholesterol in the pathogenicity of these gallbladder diseases.

Table 1 compiles the key points of nutritional factors related to canine gallbladder diseases.

## 7. Conclusions

In conclusion, nutritional factors appear to play a crucial role in the pathogenesis, prevention, and even treatment of gallbladder diseases such as mucocele and cholelithiasis. Specific dietary interventions, such as the use of omega-3 fatty acids, proteins, and fibers, can significantly influence biliary health. Dietary recommendations include complete diets with adequate levels of vitamins and proteins, particularly methionine and tryptophan, as well as moderate fat and cholesterol content.

## Figures and Tables

**Table 1:** Key points of nutritional factors related to canine gallbladder diseases.

Nutritional Impact: Nutrition plays a crucial role in managing and preventing these diseases. Factors such as dietary fat content, fiber supplementation, omega-3 fatty acids, and specific amino acids (methionine and tryptophan) are highlighted for their influence on bile composition and gallbladder motility. Dietary Management: High-fat and cholesterol-rich diets can worsen gallbladder diseases, while low-fat, low-cholesterol diets with high fiber and omega-3 supplementation may help prevent and manage these conditions. Essential Amino Acids: Methionine and tryptophan are critical for gallbladder health, with methionine playing a protective role against gallstone formation by modulating bile acid composition. Micronutrients: Vitamins such as vitamin D, riboflavin, niacin, and pantothenate may be involved in the pathogenesis of gallbladder diseases, with vitamin D deficiency potentially linked to the progression of mucocele. Feeding plan: Regular, frequent meals without prolonged fasting are crucial for maintaining gallbladder health in dogs, as this stimulates bile flow and prevents the concentration and thickening of bile, thereby reducing the risk of gallbladder diseases such as mucocele and cholelithiasis.

## Data Availability

No new data were created or analyzed in this study. Data sharing is not applicable to this article.
